# N^6^-methyladenosine-modified circ_0006168 promotes epithelial mesenchymal transition via miR-384/STAT3/Snail axis in esophageal squamous cell carcinoma

**DOI:** 10.7150/jca.97533

**Published:** 2024-07-16

**Authors:** Guandi Wu, Qin Hou, Zhe Liu, Zejin Pu, Lingfei Wu

**Affiliations:** 1 Medical Faculty Heidelberg, Heidelberg University, Heidelberg 69120, Germany.; 2 Department of Gastroenterology, Second Affiliated Hospital, Shantou University Medical College, Shantou 515041, Guangdong, China.

**Keywords:** esophageal squamous cell carcinoma, hsa_circ_006168, IGF2BP2, N^6^-methyladenosine, METTL3, miR-384

## Abstract

Circular RNAs (circRNAs) are involved in the pathogenesis of esophageal squamous cell carcinoma (ESCC). This study aimed to explore the mechanisms of aberrant expression and functions of circ_0006168 in ESCC. In this study, real-time qPCR and fluorescence in situ hybridization (FISH) are adopted to estimate the expression and localization of circ_0006168 in cancer tissues and cells. Methylated RNA immunoprecipitation (MeRIP) was performed to detect the N^6^-methyladenosine (m^6^A) modification of circ_0006168. Gain- and loss-of-functions of circ_0006168 were performed to identify its role in ESCC progression. RNA-binding protein immunoprecipitation (RIP) was used to detect the interaction of circ_0006168 with IGF2BP2. Luciferase reporter assay and RIP are used to confirm the circ_0006168/miR-384/STAT3 ceRNA network. Our results showed that the expression of circ_0006168 was upregulated in ESCC tissues and cells. METTL3-mediated m^6^A modification increased the expression of circ_0006168 via IGF2BP2-dependent way in TE-1 cells. Circ_0006168 promoted cell proliferation, migration, invasion, cell cycle progression and inhibited cell apoptosis, while knockdown of circ_0006168 had the reverse effects. Mechanistically, circ_0006168 acted its functions via miR-384/STAT3/Snail axis in TE-1 cells. In conclusion, circ_0006168 is upregulated in ESCC and m^6^A methylation increased its expression via IGF2BP2. CircRNA_0006168 promotes cell migration, invasion by regulating EMT via miR-384/STAT3/Snail axis in ESCC.

## Introduction

Esophageal cancer (EC) is one of the most common cancer types worldwide. The number of newly diagnosed cases and mortalities associated with EC ranked 7^th^ and 6^th^ , respectively, among all tumors worldwide 1. EC can be divided into esophageal squamous cell carcinoma (ESCC) and esophageal adenocarcinoma, with ESCC accounting for ~90% of all EC cases 2. The incidence rate of EC is higher in East Asia, South Central Asia, East Africa and South Europe. In China, particularly Shantou, Southern Fujian and Taihang Mountains in Guangdong are high-incidence areas 3. Despite the rapid development of early diagnostic techniques, the overall prognosis of EC remains poor 4, 5. Thus, there is an urgent need to explore the mechanism of tumorigenesis and potential therapeutic methods for ESCC.

Circular RNAs (circRNAs) are produced by reverse splicing of the exon of the precursor mRNA and are members of the non-coding RNA family. The potential effects of circRNAs in cancer progression have been widely explored 6, 7. CircRNAs play important functions in EC 8, breast cancer 9 and other cancer types 10-12. CircRNA is a single-stranded RNA with a closed loop structure that lacks the 5' cap and the 3' polyadenylated tail, and is not easily degraded by RNase R 13, 14. Due to their special circular structures, circRNAs are able to perform important biological roles by binding to microRNAs (miRNAs or miRs) 15, regulating transcription 16, interfering with splicing, and being translated into proteins and peptides 17.

*Homo sapiens* (hsa)_circ_0006168 (circ_0006168), with a total length of 395 bp, is a circRNA composed of exons 2, 3 and 4 from the CCR4-NOT transcription complex subunit 6 like (CNOT6L) gene on chromosome 4 18. Recent studies have shown that circ_0006168 is highly expressed in ESCC cells 18, 19. It has been reported that circ_0006168 acts as an oncogene, and increases cell proliferation and metastasis via miR-100 and mTOR in ESCC 18. Circ_0006168 has also been reported to promote ESCC via miR-516b-5p/X-box-binding protein 1 in ESCC 20. Qu *et al* 21 reported that circ_0006168 enhanced Taxol resistance via miR-194-5p/Jumonji domain containing 1C in ESCC.

Epithelial-mesenchymal transition (EMT) refers to a process where polarized epithelial cells are transformed into mesenchymal cells. EMT *is* a multi-step process that leads to reduced cell adhesion, and enhanced cell migration and invasion abilities, which is accompanied by decreased levels of classic epithelial markers (E-cadherin) and increased levels of mesenchymal markers (Snail, vimentin and N-cadherin) 22. EMT is associated with tumor progression and metastasis 23. To date, however, whether circ_0006168 is involved in EMT remains unknown.

miRNAs are non-coding single stranded RNA molecules with a length of 22-25 nucleotides encoded by endogenous genes 24. These small miRNAs usually target ≥1 mRNAs, and regulate gene expression by inhibiting or blocking the process of translation of target mRNAs 25. Previous studies have shown that miR-384 plays an important role in the EMT-mediated regulation of gastric cancer and nasopharyngeal carcinoma 26, 27. In the present study, bioinformatics software predictive analysis revealed binding sites between circ_0006168 and miR-384, indicating that circ_0006168 may regulate downstream genes by binding to miR-384.

N^6^-methyladenosine (m^6^A), the most abundant modification in eukaryotic RNAs, modulates RNA stability, decay, maturation, splicing, translation and export 28. m^6^A RNA methylation has a consensus motif RRACH (R = A or G, H = A, C or U). m^6^A on RNA is dependent on methyltransferases named 'writers', demethylases named 'erasers' and binding proteins named 'readers'. Methyltransferase like 3 (METTL3) is the core 'writer' protein 28. m^6^A plays important roles in RNA processing by recruiting specific 'reader' proteins. Insulin-like growth factor 2 mRNA-binding protein 2 (IGF2BP2), an important 'reader', has been shown to be able to increase the stability of RNAs with m^6^A methylation 29. However, whether m^6^A modification is involved in the regulation of circ_0006168 expression remains unclear. The molecular epitranscriptomic events in the regulation of circRNAs in the pathogenesis of ESCC are largely unknown.

In our recent study, increased expression levels of circ_0006168 were found in ESCC tissues and cells. Ectopic circ_0006168 increased the abilities of cell migration and invasion via EMT, and circ_0006168 sponged miR-384 to upregulate STAT3. More importantly, our group found that circ_0006168 was upregulated via an m^6^A-IGF2BP2-dependent manner and promoted EMT via the miR-384/STAT3/Snail signaling axis in ESCC.

## Materials and methods

### Patients and human esophageal tissue specimens

A total of 39 pairs of ESCC and adjacent non-tumorous tissues were collected from February 2016 to December 2019 at the Second Affiliated Hospital of Shantou University Medical College (Shantou, China). Radiotherapy or chemotherapy was not used in these patients before operation. The histological diagnosis of all tissues was independently confirmed by two pathologists. Differentiation grade, lymph node status and TNM stage were classified according to the 7^th^ edition UICC/AJCC TNM classification. The clinical characteristics of the patients are shown in Table 1 (age range, 35-78 years): The tissue samples were stored at -80°C immediately after adding 1 ml TRIzol^®^ reagent for RNA extraction. All patients signed informed consents before operation. All research protocols were approved by the Ethics Committee of the Second Affiliated Hospital of Shantou University Medical College (No.2020-95) and were performed following the Declaration of Helsinki.

### Cell culture

The Eca-109, KYSE-30, KYSE-450 and TE-1 ESCC cell lines, and the Het-1A normal esophageal epithelial cell line, were obtained from the Cell Bank and Stem Cell Bank, Shanghai institute for Biological Sciences, Chinese Academy of Sciences. These cell lines were cultured in DMEM (Gibco; Thermo Fisher Scientific, Inc.) supplemented with 10% (v/v) fetal bovine serum (Gibco; Thermo Fisher Scientific, Inc.), penicillin (100 U/ml) and streptomycin (100 μg/ml) at 37°C with 5% CO_2_.

### Plasmid constructs and lentiviral transduction

A lentiviral vector (Lv) encoding circ_0006168 (Lv-circ_0006168) was designed and synthesized by OBiO Technology (Shanghai) Corp., Ltd. Fragments of circ_0006168 were cloned into pLenti-EF1-EGFP-F2A-Puro-CMV-L-circRNA-WPRE (Obio Technology, Shanghai, China) to construct the lentivirus vector. The correct insertions were confirmed by DNA sequencing and the vector contained enhanced green fluorescent protein (GFP) as the detectable marker. The vector contains a specific sequence that ensures the expression of the RNA in its circular form via RNA splicing 30. An ectopic expression plasmid carrying METTL3 was also obtained (Obio Technology, Shanghai, China). Next, the lentivirus vector and auxiliary plasmid liposomes were transfected into 293T cells. After transfection, the supernatant was collected at 48 h, centrifuged at 75,000 × g for 90 min, re-suspended and filtered through 0.45 µm filters (EMD Millipore, Billerica, MA, USA). Infection efficiency was determined by GFP immunofluorescence. Circ_0006168 lentiviral expression vector conferred both green fluorescence and puromycin resistance. After 48 h, puromycin (2.0 μg/ml) was added to the culture medium, and the positive stably transduced cells were screened as our previously described 31.

Based on the mRNA sequence of circ_0006168 and METTL3, three short hairpin RNAs (shRNAs) targeting circ_0006168, named sh-circ_0006168#1, sh-circ_0006168#2 and sh-circ_0006168#3, as well as sh-METTL3 and a negative control (NC) plasmid vector (named sh-NC) were synthesized and constructed by OBiO Technology (Shanghai) Corp., Ltd. The sequences for shRNA targeting circ_0006168 and METTL3, as well as small interfering (si)RNA targeting METTL3 (si-METTL3) and IGF2BP2 (si-IGF2BP2) are listed in Table 2.

For rescue experiments aimed to verify whether circ_0006168 promoted cell proliferation via the miR-384/STAT3/Snail axis, TE-1 cells were transfected alone with Lv-circ_0006168 or Lv-NC, or co-transfected with miR-384 mimics or NC mimics. In addition, TE-1 cells were transfected alone with sh-circ_0006168 or sh-NC, or co-transfected with miR-384 inhibitor or NC inhibitor.

### Bioinformatics analysis

Circular RNA Interactome (https://circinteractome.irp.nia.nih.gov/index.html) was used to predict the target of circ_0006168. TargetScan 7.2 (https://www.targetscan.org/vert_72) was performed to predict the targets of miR-384. The Cancer Genome Atlas Program (https://www.cancer.gov/ccg/research/genome-sequencing/tcga), Genotype-Tissue Expression data (http://www.commonfund.nih.gov/GTEx) and Gene Expression Profiling Interactive Analysis (http://gepia.cancer-pku.cn) were used to determine the expression level of IGF2BP2 in EC.

### Reverse transcription-quantitative PCR (RT-qPCR)

For RNA extraction, ESCC tissues and cell lines were harvested. TRIzol^®^ reagent (Invitrogen; Thermo Fisher Scientific, Inc.) was used to extract total RNA. cDNA synthesis was performed with Takara RT kit (Takara Biotechnology Co., Ltd.). Next, qPCR was conducted using SYBR Green PCR Master Mix (Promega Corporation) in an ABI 7300 Real-Time PCR System (Applied Biosystems; Thermo Fisher Scientific, Inc.). The first denaturation step was 10 min at 95°C. Afterwards the following thermocycling protocol was utilized for 40 cycles: 95°C for 15 sec, 60°C for 30 sec, and 72°C for 30 sec. The divergent primers used to detect the expression of circ_0006168 were designed to span the circRNA backsplice junction sequence, which can specifically amplify circRNAs and not their counterpart linear RNA. The primer sequences are listed in Table 2. GAPDH and U6 (Rnu6-1) were used as internal controls for normalization. The 2^-ΔΔCq^ method was used to calculate the relative expression level of each target gene 32.

### RNase R treatment

RNase R is a 3' to 5' exoribonuclease that degrades linear RNAs without influencing circRNAs 33. A total of 2 μg RNA was incubated with or without 6 U RNase R (Qiagen GmbH) for 1 h at 37°C. Next, the expression levels of circ_0006168 and CNOT6L mRNA were determined via RT-qPCR.

### Cell viability assessment

Transfected or untransfected TE-1 cells (5x10^3^ cells/well) were plated into 96-well plates (Corning, Inc.). After incubation for 24, 48 and 72 h, 10 μl Cell Counting Kit (CCK)-8 medium (Beyotime Institute of Biotechnology) was added to every well and incubated at 37°C. At the indicated time points, the absorbance was quantified at 450 nm with a microplate reader (Thermo Fisher Scientific, Inc.).

### Cell cycle assay

TE-1 cells (1x10^5^ cells/well) were harvested, washed with ice-cold PBS, and fixed with 70% ethanol at 4°C overnight. Next, cells were treated with RNase A (KeyGen Biotech, China) at 37°C for 30 min, followed by staining with propidium iodide (Nanjing KeyGen Biotech Co., Ltd.) at 4°C in the dark for 30 min. Finally, cell cycle distribution was determined by BD CellQuest™ Pro software (version 5.2.1; BD Biosciences) and the percentage cells with apoptosis per group were calculated.

### Colony formation assessment

Untransfected or transfected for 24 h TE-1 cells (5×10^2^ cells) were seeded into 6-well plates and cultured for 2 weeks. After visible colonies were formed, the cells were fixed with 4% formaldehyde (Wuhan Servicebio Technology Co., Ltd.) at 37°C for 20 min. Cells were then stained with 0.1% crystal violet (Sangon Biotech, Co., Ltd.) solution for 20 min at room temperature and imaged using a light microscope (magnification, x40; Olympus CX31; Olympus Corporation, Tokyo, Japan).

### Wound healing assessment

Transfected or untransfected TE-1 cells were cultured and marked with a line at the bottom of the plate. A sterile pipette tip was then used to scratch the cell monolayer. Next, serum-free medium was added, following 48 h incubation at 37°C, images were obtained using a light microscope (magnification, x100; Olympus CX31; Olympus Corporation) and analyzed using ImageJ (version 1.46; National Institutes of Health).

### Transwell assay

For invasion assay, TE-1 cells (1x 10^5^ cells/well) were seeded in a 24-well Transwell upper chamber (8-µm pore size,Corning Inc.) pre-coated with Matrigel (BD Biosciences) 37°C for 24 h and cultured in DMEM without FBS. For the migration assay, TE-1 cells (1x 10^5^ cells/well) were seeded in the upper chamber without pre-coated Matrigel and cultured in DMEM without FBS. A total of 600 µl DMEM supplemented with 10% FBS (Thermo Fisher Scientific) was added to the lower chamber. Following 24 h incubation at 37°C, the migrated or invaded cells were fixed with 4% paraformaldehyde for 15 min at 37°C and stained with 0.1% crystal violet for 3 min at room temperature. Six randomly selected fields were observed with a light microscope at 200× magnification (Olympus Corporation)) for counting.

### Fluorescence in situ hybridization (FISH)

Biotin-labeled probes for circ_0006168 and FISH Kit (Exon Biotechnology Co. Ltd, Guangzhou, China) were used following the manufacturer's instructions. Circ_0006168 was visualized using the Avidin-AP antibody (1:10, Guangzhou Exon Biotechnology Co. Ltd), dyed with DAPI and finally observed under a fluorescence microscope (Nikon80i; Nikon Corporation).

### Subcellular fractionation assay

Cytoplasmic and nuclear fractions of TE-1 cells were collected using the Nuclear/Cytoplasmic Isolation kit (Norgen Biotek Corp.). U6 was used as a control in the nucleus, while GAPDH was used as a control in the cytoplasm.

### RNA immunoprecipitation (RIP) assay

Magna RIP RNA-Binding Protein Immunoprecipitation Kit (MilliporeSigma; Merck KGaA) was used to analyze the specific binding between circ_0006168 and IGF2BP2. TE-1 cells were collected and lysed in RIPA buffer. Cell lysates were then immunoprecipitated with antibodies against IGF2BP2 (cat. no. 11601-1-AP; Proteintech Group, Inc.), argonaute-2 Ago2 (cat. no. ab186733; Abcam), STAT3 (cat. no. 10253-2-AP; Proteintech Group, Inc.) or control IgG (cat. no. Ab172730; Abcam) . The expression levels of co-precipitated RNAs were assessed via RT-qPCR.

### Methylated RNA immunoprecipitation-RT-qPCR (MeRIP-RT-qPCR)

MeRIP was carried out to determinate the m^6^A levels of circ_0006168. Total RNA was first fragmented and then anti-m^6^A antibody (cat. no. ab208577; Abcam) or control IgG (cat. no. Ab172730; Abcam) was used in RIP Buffer (MilliporeSigma; Merck KGaA). RNAs were then extracted and RT-qPCR was conducted as aforementioned.

### Cell apoptosis detection

In total, 5x10^5^ cells were used per well in a 6-well plate. Cells were harvested and washed twice with PBS, then 500 µl of cell suspension in binding buffer was transferred to a 5-ml falcon tube. Next, 5 μl FITC-conjugated annexin V and 5 μl PI were added were used to stain the cells at room temperature for 30 min following the manufacturer's protocol. The percentage of apoptotic cells in each group was determined with a Cell apoptosis was analyzed using a BD FACSCalibur™ flow cytometer (Becton, Dickinson and Company) and WinMDI software (version 2.5; Purdue University Cytometry Laboratories) was used to analyze the data.

### Dual-luciferase reporter assay

Wild-type (WT) circ_0006168 sequence and 3'-untranslated region (UTR) of STAT3 were cloned into a pmirGLO vector (Promega Corporation). Mutant (MUT) reporter vectors without binding sites were also constructed. TE-1 cells were seeded into a 24-well plate the day before transfection at a density of 4x10^3^ cells/well. Next, circ_0006168-WT/MUT or STAT3-WT/MUT were co-transfected with miR-384 mimic into TE-1 cells with Lipofectamine^®^ 3000 (Invitrogen; Thermo Fisher Scientific, Inc.). Luciferase activity was then estimated with Dual-Luciferase Reporter Assay System (Promega Corporation) on a Turner BioSystems Instrument (Turner Designs).

### Western blotting

Proteins were extracted from TE-1 cells as previously described 31. Cells were washed with ice-cold PBS, harvested and lysed in RIPA buffer supplemented with protease inhibitor cocktail. Cell lysates were centrifuged at 12,000 rpm at 4°C for 10 min. The supernatant was boiled for 5 min and subjected to 10% SDS-PAGE and then transferred to PVDF membranes (MilliporeSigma; Merck KGaA). Next, 5% non-fat milk was used for blocking, and the membranes were then incubated with specific primary antibodies against METTL3 (1:1,000; cat. no. ab195352; Abcam), IGF2BP2 (1:8,000; cat. no. 11601-1-AP; Proteintech Group, Inc.), STAT3 (1:8,000; cat. no. 10253-2-AP; Proteintech Group, Inc.), phosphorylated (p)-STAT3 (Tyr705; 1:1,000; cat. no. ab76315; Abcam), E-cadherin (1:2,000; cat. no. ab40772; Abcam), vimentin (1:2,000; cat. no. ab92547; Abcam), Snail (1:2000; cat. no. ab82846; Abcam) and GAPDH (1:2,000; cat. no. ab37168; Abcam, as loading control) in TBS-Tween 20. The next day, the membranes were washed three times and incubated with a secondary antibody [HRP-conjugated goat anti-rabbit IgG (1:10,000; cat. no. ab205718; Abcam)] in the dark for 1 h, and visualized with an enhanced chemiluminescence reagent (MilliporeSigma; Merck KGaA). Bands were visualized with an ECL detection system (Thermo Scientific, Waltham, MA, USA). Protein expression was analyzed by the Quantity One software (Bio-Rad, Hercules, CA, USA) and normalized to that of GAPDH.

### Statistical analysis

GraphPad Prism 6.0 (GraphPad Software; Dotmatics) and SPSS 21.0 software (IBM Corp.) were used for statistical analyses. All experiments were repeated ≥3 times independently, and the data are presented as the mean ± SD. Two-sided paired Student's t-test was used to compare differences between two groups when data showed normal distribution, while one-way ANOVA test followed by Tukey's post hoc test was used to assess the differences among multiple groups. Pearson's correlation coefficient analysis was applied to calculate the correlation between the expression of circ_0006168 and miR-384 in ESCC tissues. P<0.05 was considered to indicate a statistically significant difference.

## Results

### Circ_0006168 expression, molecular structure and intracellular localization

**Figure 1:**
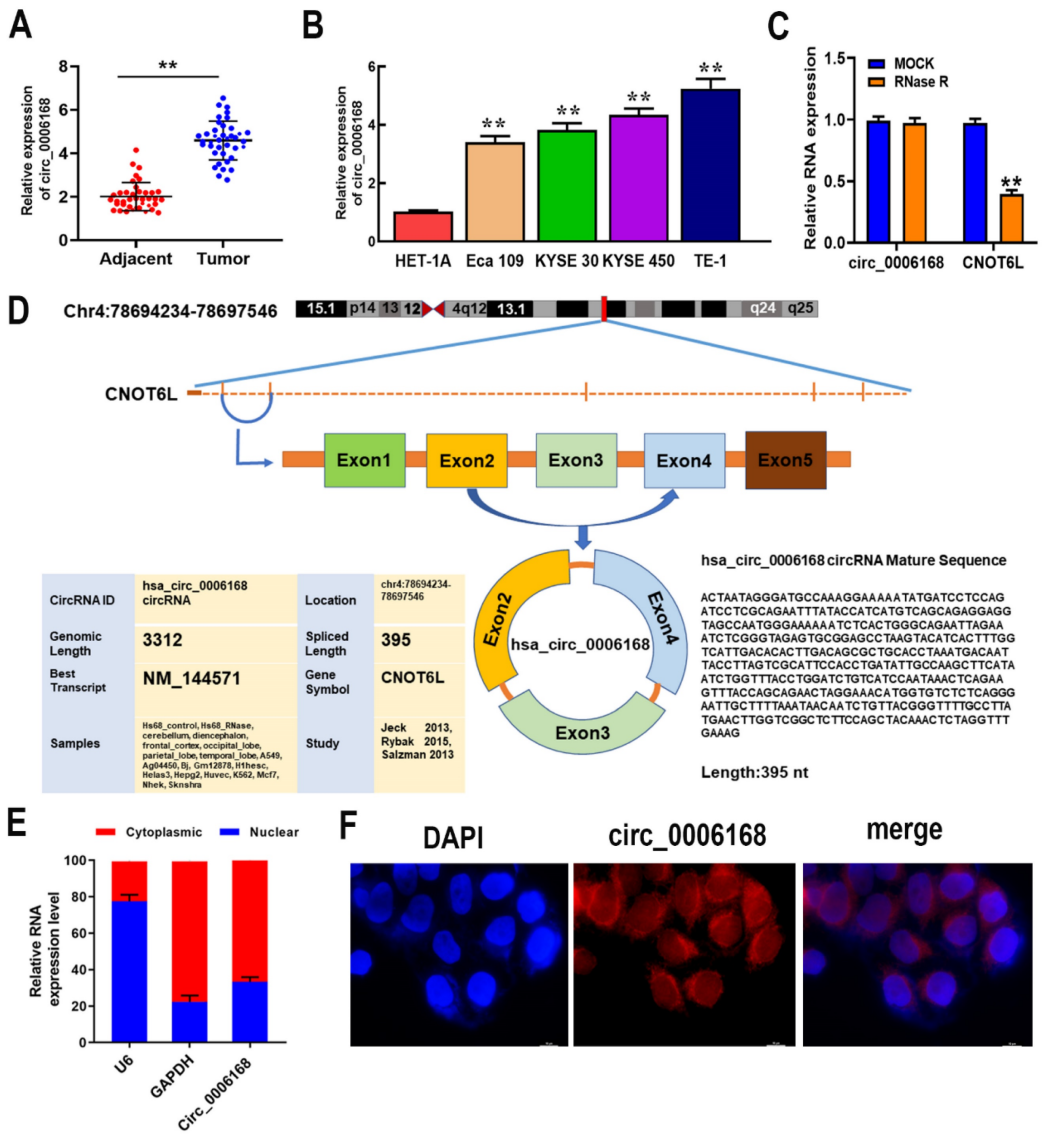
** Relative expression of circ_0006168 in ESCC tissues/cell lines and its clinical significance.** (**A**) The relative expression of circ_0006168 in ESCC and paired adjacent normal tissues was evaluated by RT-qPCR. (**B**) The relative expression of circ_0006168 in ESCC cells (Eca-109, KYSE-30, KYSE-450 and TE-1) and human normal esophageal epithelial cells (HET-1A) was detected by RT-qPCR. (**C**) CircRNAs in TE-1 cells were enriched by RNase R treatment. The expression levels of linear and circRNAs in cells were analyzed. (**D**) Diagram of circ_0006168. (**E**) The relative expression of circ_0006168 in the nucleus and cytoplasm of TE-1 cells was analyzed. U6 was used as a nuclear control and GAPDH was used as a cytoplasmic control. (**F**) Fluorescence in situ hybridization was performed to detect the intracellular distribution of circ_0006168 in TE-1 cells (magnification, x1,000; scale bar, 10 µm). TE-1 cells were co-stained with lentiviral vector-circ_0006168, HRP-conjugated anti-digoxin antibody (red) and DAPI (nucleus, blue). Data are presented as the mean ± SD (n=39). Data are expressed as the mean ± SD of three independent experiments. *P<0.05, **P<0.01 in two-tailed paired Student's t-test. ESCC, esophageal squamous cell carcinoma; RT-qPCR, reverse transcription-quantitative PCR; circ, circular.

To explore the clinical and pathological significance of circ_0006168, circ_0006168 expression in ESCC tissues we detected, and significantly upregulated circ_0006168 expression was found compared with adjacent non-tumor tissues (Fig. 1A). High expression of circ_0006168 was positively correlated with histological grade, TNM stage and lymphatic metastasis (Table 1). Compared with normal esophageal cells, all EC cells showed significantly higher levels of circ_0006168 expression, and TE-1 cells exhibited the highest expression of circ_0006168 (Fig. 1B).

To investigate RNA structure, TE-1 cells were treated with RNase R to digest linear RNA and enrich circRNAs. After RNase R treatment, the mRNA expression of CNOT6L was significantly downregulated, while the expression of circ_0006168 was not affected (Fig. 1C). The results confirmed that circ_0006168 had a cyclic structure. Circ_0006168 is located in chromosome 4: 78694234 -78697546, which is spliced from the CNOT6L gene with an ultimate length of 395 nt (Fig. 1D). Furthermore, nuclear and cytoplasmic fractionation analyses validated that circ_0006168 was mainly expressed in the cytoplasm, with a small proportion was expressed in the nucleus (Fig. 1E), which was also confirmed by FISH assay (Fig. 1F). These results collectively indicated that circ_0006168 was an abundant and stable circRNA, and its upregulation may result in carcinogenic effects on ESCC.

### METTL3-mediated m^6^A modification on circ_0006168 increases the stability and expression of circ_0006168 via the m^6^A reader protein IGF2BP2

**Figure 2:**
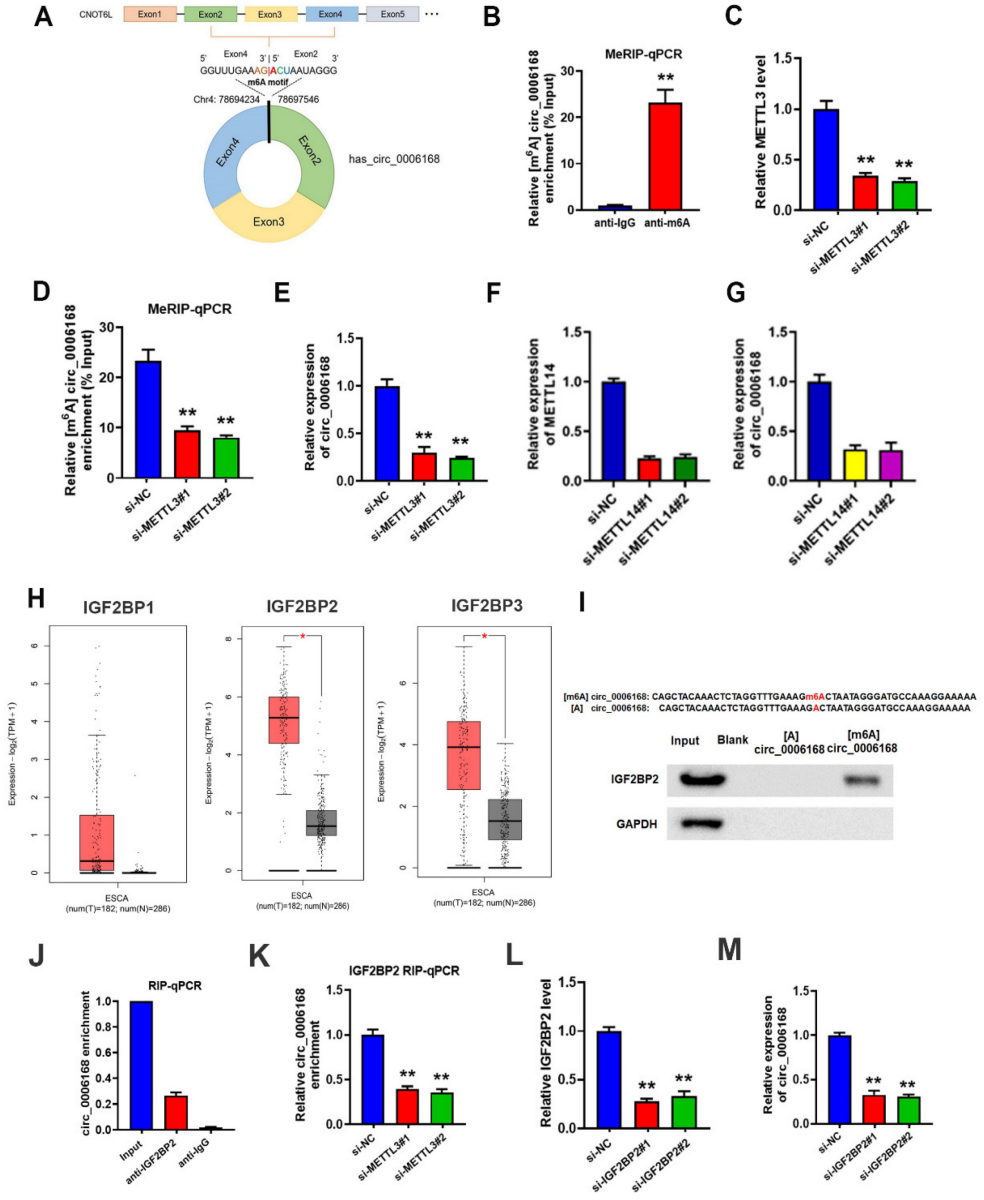
** METTL3-mediated m^6^A modification increased the stability and expression of circ_0006168 via the m^6^A reader protein IGF2BP2.** (**A**) m^6^A consensus motif at the junction site of circ_0006168. (**B**) MeRIP-qPCR analysis of circ _0006168 in TE-1 cells. (**C**) The efficiency of METTL3 knockdown was determined by RT-qPCR. (**D**) MeRIP-qPCR analysis was used to detect the m^6^A level of circ_0006168 in control and METTL3-silenced TE-1 cells. (**E**) RT-qPCR was used to detect the circ_0006168 level in control and METTL3-silenced TE-1 cells. (**F**) The efficiency of METTL14 knockdown was determined by RT-qPCR. (**G**) RT-qPCR was used to detect the circ_0006168 level in control and METTL14-silenced TE-1 cells. (**H**) IGF2BP1, IGF2BP2 and IGF2BP3 expression levels in esophageal cancer using Gene Expression Profiling Interactive Analysis 2. (**I**) RNA pull-down using 50-nt biotin-labeled m^6^A methylated or unmethylated circ_0006168 probes, followed by western blotting. (**J**) The interaction between IGF2BP2 and circ_0006168 was detected by RIP-qPCR. (**K**) The effect of silencing METTL3 on the interaction between IGF2BP2 and circ_0006168 was determined by RIP-qPCR. (**L**) The efficiency of silencing IGF2BP2 was determined by RT-qPCR. (**M**) The circ_0006168 levels in control and IGF2BP2-silenced TE-1 cells were detected by RT-qPCR. Data are expressed as the mean ± SD of three independent experiments. *P<0.05, **P<0.01 in two-tailed Student's t-test. METTL3, methyltransferase like 3; circ, circular; IGF2BP2, insulin-like growth factor 2 mRNA-binding protein 2; MeRIP; methylated RNA immunoprecipitation; RT-qPCR, reverse transcription-quantitative PCR; m^6^A, N^6^-methyladenosine.

The molecular mechanisms of circ_0006168 upregulation in TE-1 cells were investigated. An m^6^A consensus motif was found at the junction site of circ_0006168 (Fig. 2A). Since m^6^A can affect RNA stability, the present study aimed to clarify whether this m^6^A modification played a role in the stabilization of circ_0006168. As shown in Fig. 2B, the level of m^6^A was significantly increased, and the role of m^6^A modification in circ_0006168 was validated in TE-1 cells by meRIP-qPCR assay.

Next, two METTL3 interference series targeting m^6^A methyltransferase were constructed, named small interfering (si)-METTL3#1 and si-METTL3#2. The results corroborated that both si-METTL3#1 and si-METTL3#2 exhibited significant METTL3 silencing effects in TE-1 cells (Fig. 2C). The results of meRIP-qPCR assay further confirmed decreased m^6^A levels on circ_0006168 in METTL3-silenced TE-1 cells (Fig. 2D). In addition, knockdown of METTL3 downregulated circ _0006168 expression (Fig. 2E). Similarly, knockdown of METTL14 led to downregulation of circ _0006168 expression (Fig. 2F, G). Thus, it was confirmed that the m^6^A level on circ_0006168 was modulated by the m^6^A methyltransferase METTL3, and m^6^A modification increased the expression of circ_0006168.

It has been reported that m^6^A can influence RNA stability by 'readers' 34. Thus, the current study next explored which reader protein was involved in METTL3-mediated circ_0006168 regulation. The best-known m^6^A readers with the ability to enhance RNA stability are the members of the IGF2BP family 29, which includes IGF2BP1, IGF2BP2 and IGF2BP3. Combining The Cancer Genome Atlas Program and Genotype-Tissue Expression data using Gene Expression Profiling Interactive Analysis 2 35, it was found that IGF2BP2 expression was elevated most significantly among these three proteins in EC (Fig. 2H). Therefore, the present study next focused on IGF2BP2 and explored if IGF2BP2 was the reader protein of circ_0006168.

IGF2BP2 is an important 'reader' that can increase the stability of RNAs with m^6^A modification RNA pull-down was performed using 50-nt biotin-labeled m^6^A methylated or unmethylated circ_0006168 probes followed by western blotting. The results demonstrated that IGF2BP2 bound the m^6^A-circ_0006168 probe but not the unmethylated one (Fig. 2I). Next, RIP-qPCR was conducted to confirm the binding between circ_0006168 and IGF2BP2 protein (Fig. 2J). In addition, RIP-qPCR was also performed after METTL3 silencing, and it was found that silencing METTL3 restrained the interaction between circ_0006168 and IGF2BP2 protein (Fig. 2K). Next, IGF2BP2 was silenced in TE-1 cells (Fig. 2L), and it was found that silencing IGF2BP2 significantly downregulated circ_0006168 expression in TE-1 cells (Fig. 2M). These results showed that IGF2BP2 could recognize the m^6^A modification of circ_0006168 and thus enhance its stability. Overall, these results confirmed that METTL3-mediated m^6^A modification increased the stability and expression of circ_0006168 via the m^6^A reader protein IGF2BP2.

### Circ_0006168 promotes cell proliferation, migration and invasion in TE-1 cells

**Figure 3:**
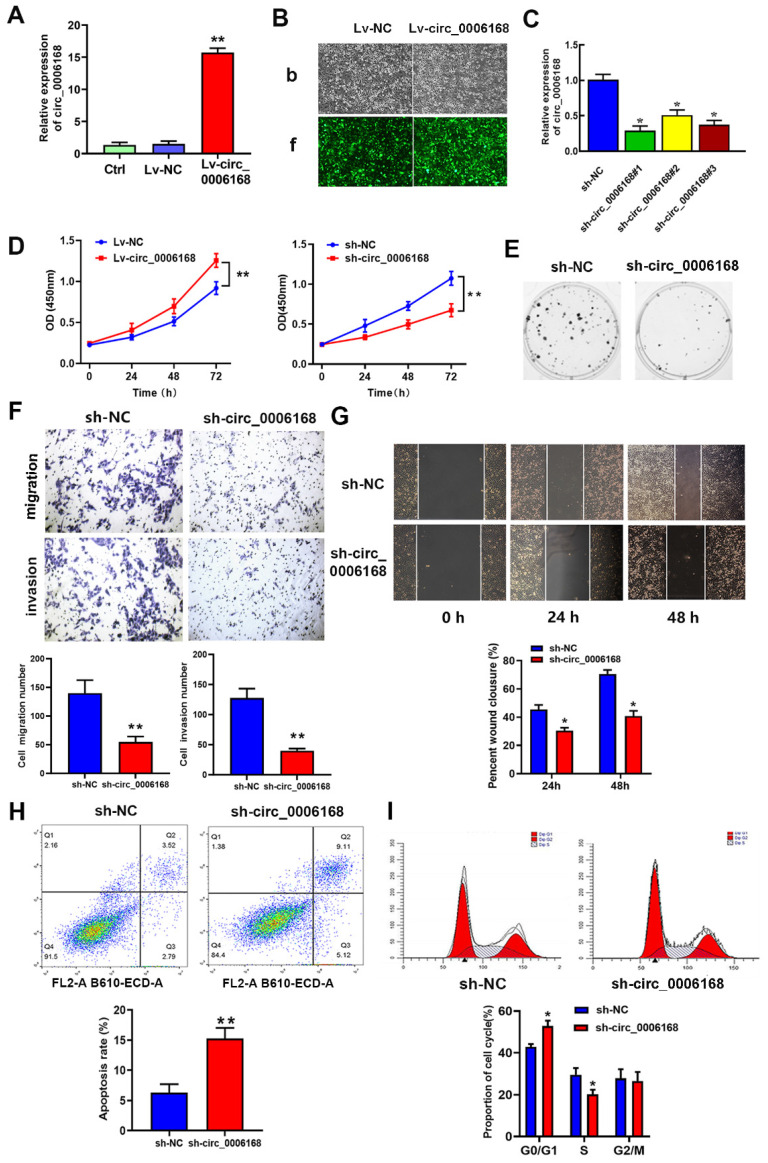
** Role of circ_0006168 in the proliferation, motility and apoptosis of TE-1 cells.** (**A**) TE-1 cells were transfected at a multiplicity of infection of 10 with Lv-circ_0006168, Lv-NC or empty Lv control, and the circ_0006168 expression level was detected by RT-qPCR. (**B**) Representative images (magnification, x100; scale bar, 100 μm) after transduction with Lvs in TE-1 cells. (B-b) Bright images (b) of TE-1 cells transfected with Lv-circ_0006168 or Lv-NC. (B-f) Fluorescence images (f) of TE-1 cells transfected with Lv-circ_0006168 or Lv-NC. (**C**) The expression levels of circ_0006168 in TE-1 cells with circ_0006168-specific shRNAs (sh-circ_0006168#1, sh-circ_0006168#2 and sh-circ_0006168#3) and sh-NC were assessed by RT-qPCR. (**D**) Cell proliferation was evaluated by Cell Counting Kit-8 assay at the indicated time points in TE-1 cells transfected with Lv- circ_0006168 and Lv-NC, or with sh-circ_0006168 and sh-NC. (**E**) Representative images of colony formation assay in TE-1 cells transfected with sh-circ_0006168 and sh-NC for 2 weeks. (**F**) The migration and invasion abilities of TE-1 cells were evaluated by Transwell assay. The number of migrated and invaded cells was determined after inoculation of transfected cells for 48 h, and cell count per field was performed (magnification, x200). (**G**) Wound healing assay was performed to detect cell migration in TE-1 cells transfected with sh-circ_0006168 or sh-NC for 48 h (magnification, x100). (**H**) Cell apoptosis in TE-1 cells transfected with sh-circ_0006168 or sh-NC was detected using flow cytometry analysis. (**I**) Knockdown of circ_0006168 caused cell cycle arrest in G1 phase, as shown by flow cytometry analysis. *P<0.05, **P<0.01 in two-tailed Student's t-test. Circ, circular; Lv, lentiviral vector; RT-qPCR, reverse transcription-quantitative PCR; NC, negative control; sh, short hairpin.

Gain- and loss-of-function experiments were performed to identify the role of circ_0006168 in TE-1 cells. A TE-1 cell line with stable overexpression of circ_0006168 was constructed, and increased expression level of circ_0006168 was confirmed in this cell line (Fig. 3A). Fluorescence imaging showed that satisfactory transduction efficiencies were achieved using Lv-circ_0006168 or Lv-NC (Fig. 3B). Besides, three interfering plasmid vectors, namely sh-circRNA#1, #2 and #3, were transfected into TE-1 cells, and they all showed significant circ_0006168 silencing effects in TE-1 cells (Fig. 3C). Compared with sh-circ_0006168#2 or sh-circ_0006168#3, sh-circ_0006168#1 showed better interference effect, and was therefore selected for subsequent experiments and named sh-circ_0006168.

The results of CCK-8 assay indicated that ectopic circ_0006168 significantly promoted cell proliferation, while knockdown of circ_0006168 efficiently suppressed cell proliferation (Fig. 3D). Colony formation assay also indicated that Lv-circ_0006168 increased colony numbers, whereas knockdown of circ_0006168 impaired colony-forming ability (Fig. 3E). Transwell and wound-healing assays further validated that knockdown of circ_0006168 suppressed cell migration and invasion (Fig. 3F and G). Flow cytometry analysis indicated that knockdown of circ_0006168 prominently increased cell apoptosis (Fig. 3H). Moreover, knockdown of circ_0006168 resulted in an increase in the proportion of G_1_-phase cells and a decrease in the proportion of S-phase cells (Fig. 3I), indicating that knockdown of circ_0006168 induced cell cycle arrest. Taken together, the present study showed that circ_0006168 not only caused cell proliferation, migration and invasion, but also promoted cell cycle progression and inhibited apoptosis in ESCC cells.

### Circ_0006168 is a miR-384 sponge

**Figure 4:**
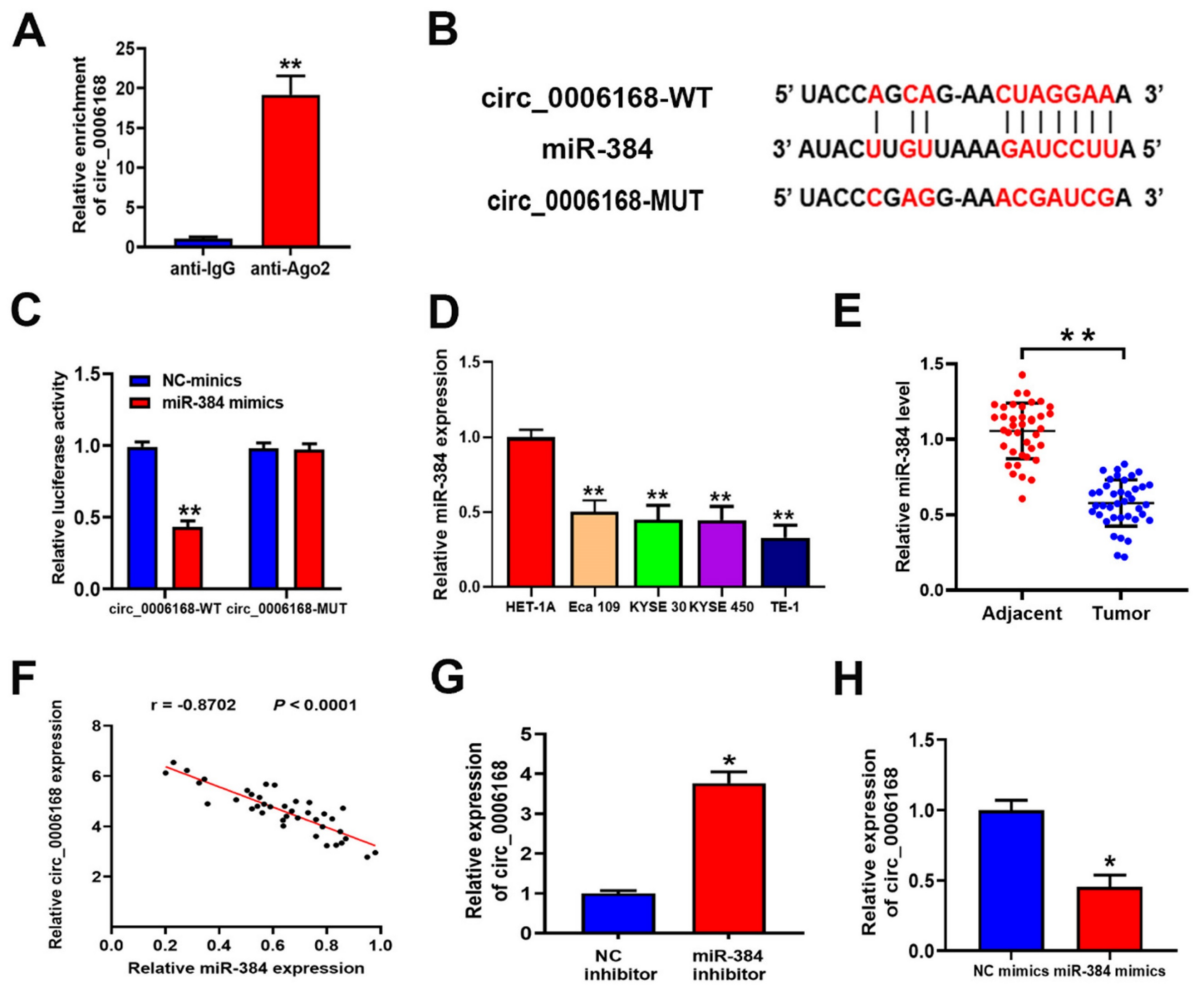
** Circ_0006168 functions as a miR-384 sponge in ESCC.** (**A**) RNA immunoprecipitation was performed to detect endogenous argounaute-2 binding to RNA. The level of circ_0006168 was detected using RT-qPCR assay. (**B**) TargetScan was used to predict the binding site between circ_0006168 and miR-384. (**C**) The luciferase activity of circ_0006168-wild-type or circ_0006168-mutant was evaluated by luciferase reporter assay upon transfection of miR-384 mimics or negative control mimics. (**D** and **E**) miR-384 expression level was detected by RT-qPCR in esophageal cancer cells and tumor tissues. (**F**) Relative expression of circ_0006168 and miR-384 in ESCC tissues, as determined by RT-qPCR assay. Pearson's correlation coefficient analysis validated the linear correlation between miR-384 and circ_0006168 expression in ESCC (r=-0.8702). (**G** and **H**) Circ_0006168 relative expression was detected using RT-qPCR after transfection with miR-384 inhibitor or mimics in TE-1 cells. *P<0.05, **P<0.01 in two-tailed Student's t-test. Circ, circular; miR, microRNA; ESCC, esophageal squamous cell carcinoma; RT-qPCR, reverse transcription-quantitative PCR.

By subcellular fractionation analysis, circ_0006168 was found to be distributed in the cytoplasm and nucleus, and was mainly localized in the cytoplasm of TE-1 cells (Fig. 1E). Ago2-RIP validated that circ_0006168 was enriched in the anti-Ago2 group (Fig. 4A), which showed that circ_0006168 was involved in RNA-induced silencing complex. Next, Circular RNA Interactome (https://circinteractome.irp.nia.nih.gov/ index.html) 36 was used to predict the target of circ_0006168, and an assumed binding site within the 3'-UTR of miR-384 was found (Fig. 4B). Dual-luciferase reporter assay verified that miR-384 mimics significantly downregulated the luciferase activity of WT circ_0006168 but not that of MUT circ_0006168, indicating that miR-384 could bind to the binding site of circ_0006168 (Fig. 4C).

The expression levels of miR-384 in different ESCC cell lines (Eca-109, KYSE-30, KYSE-450 and TE-1) are shown in Fig. 4D. Compared with the normal esophageal HET-1A cell line, the above four ESCC cell lines showed significantly lower levels of miR-384. Consistent with this, the expression level of miR-384 in ESCC tissues was also significantly lower than that in paired normal samples (Fig. 4E). Besides, a significant negative correlation was found between the expression levels of miR-384 and circ_0006168 in ESCC tissues (Fig. 4F). Knockdown of miR-384 increased the expression of circ_0006168 in TE-1 cells (Fig. 4G). On the contrary, miR-384 mimics decreased the expression level of circ_0006168 in TE-1 cells (Fig. 4H). Taken together, these results indicated that circ_0006168 acted as a miR-384 sponge in ESCC cells and tissues.

### STAT3 is the target gene of miR-384, and circ_0006168 and miR-384 regulate EMT via STAT3

**Figure 5:**
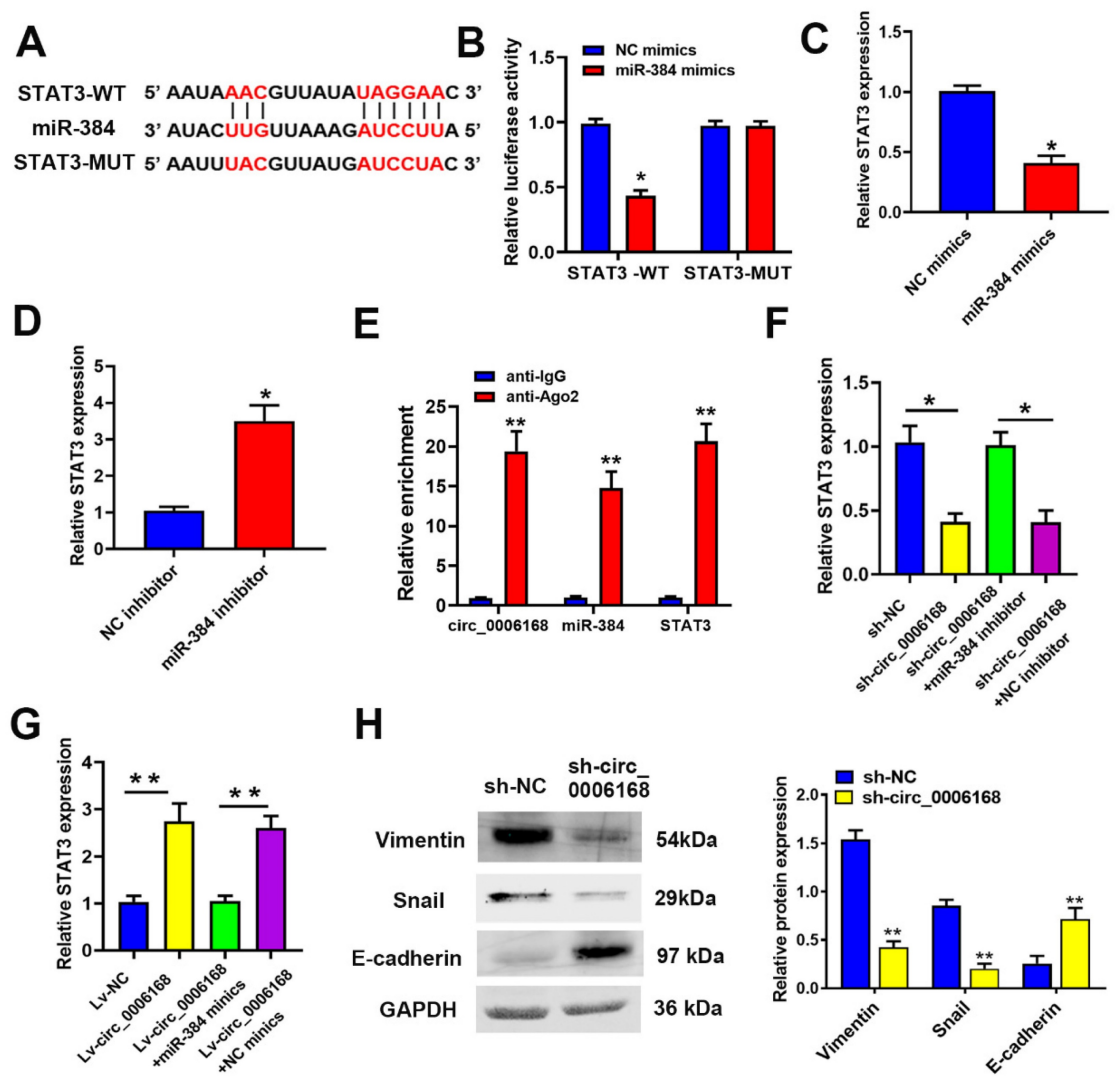
** Circ_0006168 promotes TE-1 cell proliferation via the miR-384/STAT3 axis.** (**A**) The binding site (WT or MUT type) of STAT3 to miR-384 is shown, as predicted through TargetScan. (**B**) The luciferase activity of STAT3-WT or STAT3-MUT was evaluated by luciferase reporter assay upon transfection of miR-384 mimics or NC mimics. (**C** and **D**) STAT3 expression level after miR-384 overexpression or knockdown in TE-1 cells was evaluated by RT-qPCR assay. (**E**) Co-precipitated RNAs of circ_0006168, miR-384 and STAT3 in the argonaute-2 or IgG groups were measured by RNA immunoprecipitation followed by RT-qPCR assay. (**F**) STAT3 mRNA expression levels in TE-1 cells with sh-circ_0006168 or sh-NC alone, or co-transfected with miR-384 inhibitor or NC inhibitor were detected by RT-qPCR. (**G**) STAT3 mRNA expression levels in TE-1 cells transfected with Lv-circ_0006168 or Lv-NC alone, or co-transfected with miR-384 mimic or NC mimics were detected by RT-qPCR. (**H**) The expression levels of EMT-related proteins (E-cadherin, vimentin and Snail) in TE-1 cells transfected with sh-circ_0006168 or sh-NC were assessed by western blotting. The expression levels of EMT-related proteins (E-cadherin, vimentin and Snail) in TE-1 cells transfected with sh-circ_0006168 or sh-NC were assessed by western blotting. Bar graphs represent mean protein band intensity, and relative quantity of protein was calculated after normalization to GAPDH. *P<0.05, **P<0.01 in two-tailed Student's t-test. Circ, circular; miR, microRNA; WT, wild-type; MUT, mutant; NC, negative control; RT-qPCR, reverse transcription-quantitative PCR; sh, short hairpin; Lv, lentiviral vector; EMT, epithelial-mesenchymal transition.

To explore the downstream mechanism of miR-384, TargetScan 7.2 (https://www. targetscan.org/vert_72) 37 was performed to predict the targets of miR-384. Among the potential targets, a putative binding site for miR-384 was found in STAT3 (Fig. 5A). STAT3 was selected as a potential candidate due to its crucial role in tumor growth 38-40 and EMT 41. To further confirm the direct binding of miR-384 to the 3'-UTR of STAT3 mRNA, a dual-luciferase reporter assay was applied. TE-1 cells were co-transfected with miR-384 mimic and pmirGLO vectors, and a remarkable reduction in relative luciferase activity was observed in the STAT3 WT 3'-UTR group, whereas no significant reduction was found in the STAT3 mut 3'-UTR group (Fig. 5B).

RT-qPCR assay showed that miR-384 mimics significantly decreased STAT3 expression, whereas a miR-384 inhibitor increased STAT3 expression in TE-1 cells (Fig. 5C and D). Moreover, it was confirmed via RIP assay that circ_0006168, miR-384 and STAT3 co-localized in Ago2 protein complex (Fig. 5E). Knockdown of circ_0006168 significantly decreased the mRNA expression of STAT3, which was reversed by a miR-384 inhibitor (Fig. 5F). Additionally, ectopic overexpression of circ_0006168 upregulated STAT3 mRNA expression, whereas miR-384 mimics could reverse this effect (Fig. 5G). Taken together, these results indicated that miR-384 could downregulate STAT3 expression by targeting the 3'-UTR of STAT3 mRNA in TE-1 cells., suggesting that circ_0006168 could function as a miR-384 sponge and thus upregulate STAT3 expression.

Since circ_0006168 enhanced STAT3 expression and was reported that STAT3 participated in EMT regulation 41, western blotting was used in the present study to detect the expression levels of EMT-related proteins (namely vimentin, E-cadherin and Snail). Knockdown of circ_0006168 significantly increased E-cadherin expression, and inhibited the expression of vimentin and Snail (Fig. 5H). Knockdown of circ_0006168 inhibited EMT in TE-1 cells. Since upregulation of STAT3 was triggered by circ_0006168, it was assumed that circ_0006168 could activate the STAT3-dependent EMT signaling pathway in TE-1 cells.

### Circ_0006168 promotes cell proliferation and EMT via the miR-384/STAT3 axis

**Figure 6:**
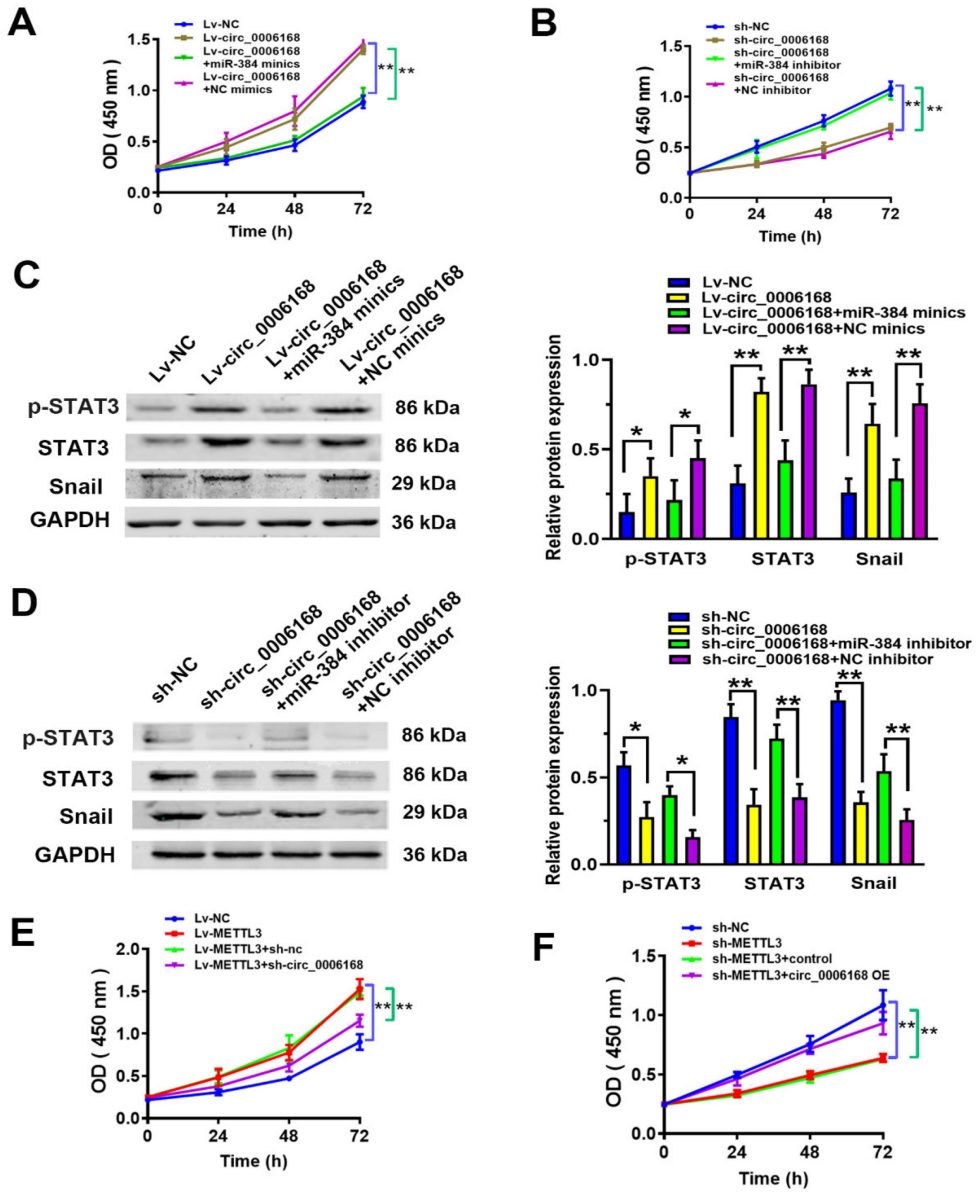
** Circ_0006168 promotes cell proliferation via the miR-384/STAT3/Snail axis in TE-1 cells.** (**A**) CCK-8 assay showed that ectopic circ_0006168 promoted cell proliferation and miR-384 mimics reversed circ_0006168-induced proliferation in TE-1 cells. (**B**) CCK-8 assay confirmed that cell proliferative ability was decreased upon knockdown of circ_0006168, which was reversed by miR-384 inhibitor. (**C**) The protein expression levels of p-STAT3, STAT3 and Snail were detected by western blotting in TE-1 cells transfected with Lv-circ_0006168 or Lv-NC alone, or co-transfected with miR-384 mimic or NC-mimics. (**D**) The protein expression levels of p-STAT3, STAT3 and Snail were detected by western blotting in TE-1 cells transfected with sh-circ_0006168 or sh-NC alone, or co-transfected with miR-384 inhibitor or NC-inhibitor. Bar graphs represent mean protein band intensity. Relative quantity of protein was calculated after normalization to GAPDH. (**E**) CCK-8 assay showed that ectopic METTL3 promoted cell proliferation, while circ_0006168 knockdown reversed METTL3-induced proliferation in TE-1 cells. (**F**) The cell proliferative ability was restricted upon METTL3 depletion, which could be reversed by circ_0006168 overexpression. *P<0.05, **P<0.01 in two-tailed Student's t-test. Circ, circular; miR, microRNA; CCK, Cell Counting Kit; p-, phosphorylated; Lv, lentiviral vector; NC, negative control; sh, short hairpin; METTL3, methyltransferase like 3.

To evaluate whether circ_0006168 functioned in ESCC proliferation by miR-384, rescue experiments were performed. The results of CCK-8 assay showed that ectopic circ_0006168 promoted cell proliferation, while miR-384 mimics reversed circ_0006168-induced proliferation in TE-1 cells (Fig. 6A). Cell proliferative ability was inhibited upon circ_0006168 depletion, which could be reversed by miR-384 inhibitor (Fig. 6B). Overexpression of circ_0006168 significantly upregulated the mRNA level of STAT3 (Fig. 5G). It has been reported that p-STAT3 can promote EMT by activating the Snail pathway 42. Thus, the effects of circ_0006168 on the protein expression of p-STAT3, total STAT3 (STAT3) and Snail were evaluated by rescue experiments. As shown in Fig. 6C, ectopic circ_0006168 enhanced p-STAT3, STAT3 and Snail expression, while miR-384 mimics partially restrained these effects. By contrast, knockdown of circ_0006168 markedly suppressed the expression of p-STAT3, STAT3 and Snail, which could also be reversed by miR-384 inhibitor (Fig. 5D). Overall, the present data demonstrated that circ_0006168 promoted ESCC proliferation and accelerated EMT progression via the miR-384/STAT3/Snail axis.

Rescue experiments were performed to explore whether the m^6^A modification functioned in ESCC proliferation by circ_0006168. The results of CCK-8 assay showed that ectopic METTL3 promoted cell proliferation, while circ_0006168 knockdown reversed METTL3-induced proliferation in TE-1 cells (Fig. 6E). Cell proliferative ability was decreased upon METTL3 depletion, which could be reversed by circ_0006168 overexpression (Fig. 6F). These results supported that the m^6^A modification functioned in ESCC proliferation by circ_0006168.

## Discussion

CircRNAs, highly conserved non-coding RNAs, play an important role in cancer development 43. Previous research has shown that circ_0006168 is significantly upregulated in EC cells 19, 20 and m^6^A modification is closely associated with the process of tumor development 28. Although these studies shed some light on the functions and mechanisms of circ_0006168, the exact role of m^6^A methylation in ESCC carcinogenesis and metastasis remains unknown.

The present study indicated that circ_0006168 was upregulated in ESCC tissues and cells, and higher levels of circ_0006168 expression correlated with aggressive histological grades and TNM stage in patients with ESCC. Ectopic expression of circ_0006168 promoted cell proliferation, migration, invasion and cell cycle progression, and inhibited cell apoptosis in TE-1 cells. These results further confirmed that circ_0006168 played an important oncogenic role in ESCC 18-20.

Next, the mechanisms of aberrant expression of circ_0006168 in ESCC were explored in the present study. Previous research has shown that m^6^A modification plays an important role in the expression and functions of circRNAs. The m^6^A modification of circIGF2BP3 confers circularization 44. YTHDC1 recognizes m^6^A modification of circNSUN2 to facilitate its export from the nucleus to the cytoplasm 45. A recent study showed that circAFF2 was a novel m^6^A-modified circRNA, and the AlkB homolog H5/YTH N^6^-methyladenosine RNA binding protein F2/circAFF2/cullin- neural precursor cell expressed developmentally downregulated gene 8 axis enhanced the radiosensitivity of colorectal cancer 46. m^6^A modification plays an important role in circ-YAP translation, resulting in YAP dephosphorylation and thus enhancing colorectal cancer liver metastasis 47. Fat mass and obesity-associated protein led to downregulation of m^6^A modification of circFAM192A and promoted gastric cancer proliferation 48. Another m^6^A-modified circRNA, circ_0124554, can promote colorectal cancer progression and radioresistance via LIM and SH3 protein 1 49. Among the m^6^A reader proteins, IGF2BP2 acts mainly by enhancing RNA stability 50, 51. Notably, the present study found a m^6^A consensus motif at the junction site of circ_0006168. The m^6^A modification of circ_0006168 was confirmed by meRIP-qPCR assay, and it was found that the m^6^A level on circ_0006168 was modulated by METTL3 and could increase the expression of circ_0006168. In the present study, 50-nt biotin-labeled m^6^A methylated or unmethylated circ_0006168 probes were synthesized, RNA pull-down assay was conducted, and it was found that IGF2BP2 bound the m^6^A-circ_0006168 probe but not the unmethylated one. Further experiments, including RIP-qPCR, demonstrated that IGF2BP2 recognized the m^6^A modification of circ_0006168, thus increasing the stability and expression of circ_0006168. The present results revealed the mechanism by which METTL3-mediated m^6^A modification increased circ_0006168 expression via an IGF2BP2-dependent manner in ESCC.

It has been reported that circRNAs are able to bind to miRNAs and inhibit the activity of miRNAs 52. Previous research shown that miR-384 may act as a tumor suppressor gene in colorectal cancer 53, oral squamous 54 and prostate cancer 55. In addition, some non-coding RNAs, such as LINC01087 56, TUG1 27, NEAT1 57 and circ_0020123 58, were verified to target miR-384 in cancer progression. The present study confirmed that miR-384 was a direct target of circ_0006168. Moreover, ectopic circ_0006168 increased cell proliferation, and miR-384 mimics could reverse the effect of circ_0006168 on cell proliferation. On the contrary, knockdown of circ_0006168 attenuated cell proliferation, which could also be reversed by miR-384 inhibitor. This evidence further confirmed that circ_0006168 served as a miR-384 molecular sponge and exerted its function via miR-384 in ESCC.

STAT3, as an oncogene, can be transported to the cell nucleus to mediate the transcription of target genes after its activation 59. STAT3 plays an important role in multiple tumors, including breast 60, prostate 61 and colon cancer 62, as well as liver carcinoma 63 and EC 38. STAT3 is involved in the tumorigenic phenotype through different ways, such as regulating cell cycle progression or regulating apoptosis-related genes 64-66. Under the stimulation of cytokines, hormones or protein kinases, phosphorylation of a specific receptor tyrosine residue (Tyr705 or Ser727) can cause sustained activation of STAT3, and thus maintain the biological characteristics and behavior of malignant tumors 67. EMT participates in biological processes, including formation, invasion and metastasis of various tumors 22. Adhesion factors, including E-cadherin, vimentin and N-cadherin, are important in the EMT process. Lack of intercellular connecting substances and differences in extracellular matrix structure lead to stronger abilities of invasion and metastasis in cancer cells. Therefore, it is of great significance to explore the regulatory mechanisms of EMT in ESCC. Our previous study confirmed that long no-coding RNA (lncRNA) MEG3 could affect EMT by regulating the GSK-3β/Snail signaling pathway in ESCC 68. Yin *et al* 69 reported that STAT3 could induce Snail expression and participate in the regulation of the EMT signaling pathway. Other EMT regulators, including zinc finger E-box binding homeobox 1/2 proteins, TGF-β, Twist and Slug, are also influenced via STAT3 signaling. Various molecules such as miRNAs, lncRNAs and circRNAs have been reported to regulate the STAT3/EMT axis 70. Although circRNAs are considered as key gene regulators in multiple cell processes, EMT-related circRNAs in ESCC still remain largely unknown. Previous studies have shown that *Homo sapiens* (hsa)_circ_0006948 71 and hsa_circ_0012563 72 promote the migration and invasion of ESCC by enhancing the EMT pathway. CircVRK1 has been reported to suppress EMT via the miR-624-3p/PTEN/PI3K/AKT signaling pathway in ESCC 73. These studies indicate the complex mechanisms of EMT-related circRNAs in ESCC. The present study confirmed that circ_0006168 increased STAT3 expression and enhanced its activation, which further increased the expression of Snail and vimentin, and also decreased E-cadherin expression in TE-1 cells. miR-384 mimics partially restrained these effects. Taken together, the current study identified a novel regulatory EMT-related axis formed by the circ_0006168/miR-384/STAT3 signaling pathway in ESCC. The migration of ESCC cells significantly decreased overall survival in patients with ESCC. Thus, important efforts should be made to explore the underlying mechanisms relevant to the EMT of ESCC cells, including EMT-related circRNAs.

In summary, the current study validated that circ_0006168 was overexpressed, which could enhance cell proliferation, invasion, migration in ESCC. Moreover, the data uncover the mechanism by which METTL3-mediated m^6^A modification increased circ_0006168 expression via an IGF2BP2-dependent manner in ESCC. Circ_0006168 functions as a competing endogenous RNA to upregulate STAT3 and promote EMT by activating the miR-384/STAT3/Snail axis. The current findings provide a novel insight to clarify the mechanisms of aberrant expression and functions of circ_0006168 in ESCC, and will improve the understanding of this potential target for ESCC therapy.

## Figures and Tables

**Table 1:** Associations between clinicopathological features and the expression of circ_0006168 in ESCC patients

Characteristics	Number of patients	Relative expression of circ_0006168		*P* value
		High	Low	
**Gender**				0.465
Male	29	17	12	
Female	10	4	6	
**Age (years)**				0.682
<60	7	3	4	
≥60	32	18	14	
**Drinking history**				0.285
Yes	11	4	7	
No	28	17	11	
**Family history of ESCC**				0.647
Yes	5	2	3	
No	34	19	15	
**Tumor size (cm)**				0.055
<3	15	5	10	
≥3	24	16	8	
**Tumor location**				0.593
Upper	7	4	3	
Middle	22	13	9	
Lower	10	4	6	
**Histologic grade**				0.008
Moderate	26	10	16	
Poor	13	11	2	
**TNM stage**				0.001
IA-IIB	17	4	13	
IIIA-IV	22	17	5	
**Lymphatic metastasis**				0.011
Negative	11	2	9	
Positive	28	19	9	

**Table 2:** Primers used for quantitative real time-PCR and other assays in this study.

Primers used for quantitative real time-PCR		
**Gene Symbol**	**Forward (5'→ 3')**	**Reverse (5'→ 3')**
Circ_0006168	CTTATGAACTTGGTCGGCTCCT	GCTACCTCCTCTGCTGACAT
U6	GCTTCGGCAGCACATATACTAA	AACGCTTCACGAATTTGCGT
GAPDH	GTCTTCACCACCATGGAGAA	TAAGCAGTTGGTGGTGCAG
miR-384	TGTTAAATCAGGAATTTTAA	TGTTACAGGCATTATGAA
STAT3	CAGCAGCTTGACACACGGTA	AAACACCAAAGTGGCATGTGA
**Oligonucleotides for short hairpin RNA**		
shRNA-NC	5'-CCTAAGGTTAAGTCGCCCTCG-3'	
sh-METTL3-NC	5'-GGAGATCCTAGAGCTATTA-3'	
sh‑circ_0006168#1	F: 5'-CCAGATCCTCGCAGAATTT-3' R: 5'-CAAAAAACCAGATCCTCGC-3'	
sh‑circ_0006168#2	F: 5'-GCTGCACCTAAATGACAAT-3' R: 5'-CAAAAAAGCTGCACCTAAA-3'	
sh‑circ_0006168#3	F: 5'-CCTGGATCTGTCATCCAAT-3' R: 5'-CAAAAAACCTGGATCTGTC-3'	
siRNA sequences		
siControl	5'-UUCUCCGAACGUGUCACGUTT-3'	
siMETTL3-#1	5'-GGAGAUCCUAGAGCUAUUATT-3'	
siMETTL3-#2	5'-GCACAUCCUACUCUUGUAATT-3'	
siMETTL14-#1	5'-GCTAAAGGATGAGTTAATA-3'	
siMETLL14-#2	5'-GCAGCACCTCGGTCATTTA-3'	
siIGF2BP2-#1	5'-CATGCCGCATGATTCTTGA-3'	
siIGF2BP2-#2	5'-GAACGAACTGCAGAACTTA-3'	
